# The role of community pharmacists in increasing access and use of self-care interventions for sexual and reproductive health in the Eastern Mediterranean Region: examples from Egypt, Jordan, Lebanon and Somalia

**DOI:** 10.1186/s12961-021-00695-0

**Published:** 2021-04-21

**Authors:** Luna El Bizri, Laila Ghazi Jarrar, Wael K. Ali Ali, Abdifatah H. Omar

**Affiliations:** 1 School of Pharmacy, Lebanese University, Hadath, Lebanon; 2 Jordan Pharmacists Association, Amman, Jordan; 3 EMROpharm Forum, FIP, Cairo, Egypt; 4 Somali Pharmacists Association, Mogadishu, Somalia

**Keywords:** Pharmacy, Pharmacists, Community, Sexual and reproductive health, Self-care intervention, Eastern Mediterranean Region, COVID-19

## Abstract

**Background:**

Self-care interventions offer a solution to support the achievement of three goals of the World Health Organization (WHO): to improve universal health coverage, reach people in humanitarian situations, and improve health and well-being. In light of implementing WHO consolidated guidelines on self-care interventions to strengthen sexual and reproductive health (SRH) in the Eastern Mediterranean Region (EMR), especially during the COVID-19 pandemic, pharmacists from four different EMR countries discussed the current SRH situation, inequality gaps, barriers to SRH service access and the pharmacist’s crucial role as a first-line responder to patients before, during and after COVID-19.

**Case presentation:**

Self-care interventions for SRH allow health care providers to serve a greater number of patients, improve progress toward universal health coverage, and reach people in humanitarian crises. In fact, these interventions can be significantly enhanced by utilizing community pharmacists as first-line health care providers. This review highlights the important role of community pharmacists in promoting self-care interventions and empowering individuals, families and communities. As a result, well-informed individuals will be authoritative in their health decisions. Exploring self-care interventions in the EMR was done through reviewing selected SRH services delivery through community pharmacists before and during the COVID-19 pandemic in Egypt, Jordan, Lebanon and Somalia. Before the COVID-19 pandemic, community pharmacists were found to be excluded from both governmental and nongovernmental SRH programmes. During the pandemic, community pharmacists managed to support patients with self-care interventions, whether voluntarily or through their pharmacy associations. This highlights the need for the health care decision-makers to involve and support community pharmacists in influencing policies and promoting self-care interventions.

**Conclusion:**

Self-care interventions can increase individuals’ choice and autonomy over SRH. Supporting community pharmacists will definitely strengthen SRH in the EMR and may help make the health system more efficient and more targeted.

## Sexual and reproductive health (SRH): an ongoing challenge in the Eastern Mediterranean Region (EMR)

### Background

In this review, four countries from the EMR are discussed: Egypt, the second most populous country in the World Health Organization (WHO) EMR (WHO 2010), with a very young population; Jordan, with an estimated total population of about 9.5 million – 37% under 15 years of age – which is renowned for its high-quality health care services (WHO 2017); Lebanon, with a population estimated at 4 million – over 80% of whom live in urban areas – which is ranked 17th out of 21 countries in health production and determinants in EMR countries; and finally, Somalia, a fragile state with 15 million Somalis (World Bank 2019) which lacks proper health facilities, while 72% of its population has no access to proper health care [1].

WHO defines sexual health as “a state of physical, emotional, mental and social well-being in relation to sexuality; it is not merely the absence of disease, dysfunction or infirmity”. [2]. Although sexual health is a vital sign of the overall health, unfortunately, numerous barriers prevent individuals’ SRH needs and concerns from being met in many regions and countries. Inequalities and disparities in access to services based on the geographical location (urban versus rural) and income/wealth are seen among individuals in low- and middle-income countries (LMICs) and need to be addressed to improve equity. Major gaps described were inequalities in access to SRH services and adequate education and information, with women and adolescents being left in the lowest two wealth quintiles [3]. More specifically, in the EMR, SRH is a public health challenge due to the high prevalence of HIV/AIDS among people who inject drugs (Egypt) and men who have sex with men (Lebanon) or among sex workers (Somalia, Egypt).

The biological and social impact of sexually transmitted diseases (STDs) and the ethical challenges, such as cultural and religious ones, are also influencing factors [4]. Improving maternal and newborn health is still a challenge for disadvantaged countries in the region [5]. In this region, SRH is still perceived as a sensitive and embarrassing taboo: discussion of sexuality with health care providers or family, especially among young people and notably unmarried girls, remains constrained; HIV-positive individuals – notably women – may hide their health status from their caregivers. This embarrassment in disclosing sexual activity may prevent people from presenting to their health facilities, and this will have profound effects on the individual, the family and the community.

Barriers to the provision of SRH services in the region include but are not limited to:

- Fear of stigma
- Loss of social status
- Shame and embarrassment
- Unreliable, disrespectful health care providers
- Lack of privacy/confidentiality within health facilities
- Inaccessibility of appropriate information, counselling and medicines

To address SRH concerns, patient barriers should be aligned with potential solutions offered by professional health care providers, and pharmacists can play a significant role. In this concept, the International Pharmaceutical Federation (FIP) – the global body representing pharmacy and pharmaceutical sciences – recommends that pharmacy organizations should support pharmacists to provide information on care options as well as logistical and financial details pertaining to health care in a way that empowers women to make the best decisions for their family and those for whom they are responsible [6]. FIP also recommends that individual pharmacists take greater responsibility in maternal, newborn and child health (MNCH), taking into account their practice responsibilities and their respective nationally defined scopes of practice [7].

## Leveraging self-care interventions for better SRH

WHO defines self-care as the ability of individuals, families, and communities to promote and maintain health, prevent diseases, and cope with an illness or a disability with or without the support of a health care provider. Self-care encompasses several issues including hygiene, nutrition, lifestyle, environmental and socioeconomic factors. Promotion of self-care is a means to empower individuals, families, and communities for informed health decision-making. It has the potential to improve the efficiency of health systems and contribute towards health equity [8].

Despite poor education and information about SRH as well as the taboo perceived when discussing sexuality and reproduction, governments in EMR countries have created and implemented programmes to promote and encourage a range of self-care interventions for SRH. For instance, the United Nations Population Fund (UNFPA) in Jordan is working towards improving young people’s ability to exercise SRH in development and humanitarian settings in the country. Under the current country programme (2018–2022), the youth programme utilizes three main approaches to empower young people to exercise their SRH rights [9]:

1. Integrating SRH curricula among youth and adolescents
2. Promoting innovative approaches for knowledge transfer on SRH and youth peace and security (YPS)
3. Advocating for inclusion of adolescent and youth SRH in national strategies and policies, including emergency preparedness plans.

The importance of community pharmacists in self-care interventions in the context of COVID-19 cannot be overstated and falls under the need to ensure and leverage the sustainability of primary health care. Furthermore, it offers an additional choice and provides the confidentiality that could protect people from being victimized, marginalized or criminalized due to lack of access to regulated quality products and interventions, as well as elective medical services, as a result of the lockdown measures. During the confinement, mainly emergency cases and operations were provided.

## The role of pharmacists in self-care interventions for SRH

**Fig. 1:**
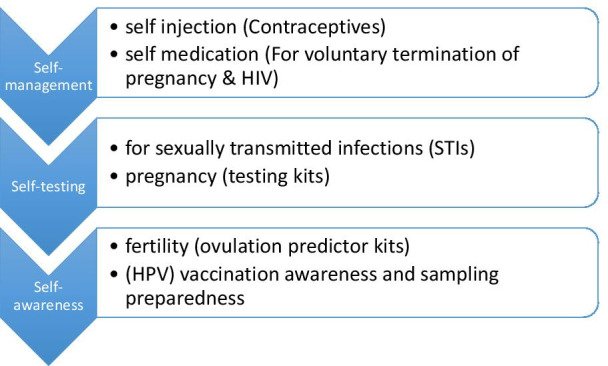
Selected self-care interventions that pharmacists deliver routinely in many EMR countries

SRH is an important need that should be targeted in the community by health care professionals, and especially by pharmacists as they serve as first-line responders to patients. They play a crucial role in the community to counsel and empower patients by providing information on and delivering self-care interventions to maximize health benefits (Fig. 1 gives examples of these interventions) (Table 1).

**Table 1:** Number of pharmacists compared to estimated population in the four studied countries

	Estimated population	Number of registered pharmacists	Number of non-registered pharmacists
Egypt	98.42 million	250 000	NA
Jordan	9.5 million	25 000	NA
Lebanon	4 million	9 000	NA
Somalia	15 million	90	310

Data collected from records of the World Bank (2018), the Jordan Pharmacists Association (JPA), and the Order of Pharmacists of Lebanon (OPL). *NA* not applicable

The total number of pharmacists in the EMR is around 460 000 [10]. With a population of 583 million in the EMR, these figures show that more than 61% of pharmacists in the EMR are present in the four countries and could imply an imbalance or shortage of pharmacists in the remaining EMR countries.

In some countries, the shortage of qualified, accredited and trained pharmacists practicing in community settings negatively affects effective pharmacy practice. In some developing countries, pharmacists prefer to work in cities rather than rural areas due to the affluent urban areas – a factor that creates a difference between the health services available in the urban and rural areas [11].

More precisely, a shortage of community pharmacists leads to a shortage of pharmacy services in general as well as a shortage in supporting patients with their self-care. In this case, the final aim, which is to promote patients to be willing to improve their own health outcomes, will not be achieved (Table 2).

**Table 2:** Key messages emerging from the 2019 Joint Statement of Policy by the International Pharmaceutical Federation (FIP) and the Global Self-Care Federation (GSCF) on Responsible and Effective Self-care [12]

*Pharmacists have a professional responsibility to:*
Support the person with evidence-based, unbiased and sound advice about self-care, the range of available treatment options, and the accurate self-identification of many self-treatable conditions
Provide each patient with holistic advice, which may include but is not limited to: diet, exercise and other lifestyle changes
Adapt communication to the individual’s level of health literacy
Encourage the individual to provide the pharmacist with information required to best assess a condition
Encourage the person to always use self-care products appropriately, safely, efficaciously and judiciously
Invite the person to speak to the pharmacist if he/she experiences unexpected/unwanted effects from the use of a self-care product
Encourage the person to proactively manage his/her own health and lifestyle and establish a strong relationship with their pharmacist
Advise the public on the benefits of consulting the pharmacist as an expert health care professional when looking to self-care
Encourage the person to advise family and friends to consult a pharmacist or other appropriate health care professionals in the case of health complaints
Help improve the awareness of the public and health care professionals to utilize self-care to address public health issues of current importance

In 2019, FIP issued a policy statement consolidating the role of pharmacists in promoting responsible and effective self-care. Access to primary health care services including self-care interventions for SRH can be better attained through community pharmacies for the following reasons [12]:

- Skilled staff: pharmacists are trained and certified to support the person with evidence-based, unbiased and sound advice about self-care.
- Resourceful: pharmacists provide a friendly environment that is also credible, acceptable, unintimidating, knowledgeable, trusted, caring, confidential and accessible.
- Enabling environment: pharmacies are already equipped to store, handle and dispense regulated quality medical products and interventions.
- National accessibility: the wide distribution of pharmacies and long operating hours provide good round-the-clock geographical access to community pharmacies.
- Reporting and feedback: pharmacist education and training enables them to capture information as needed and report back to the corresponding authority (pharmacovigilance).

The following situations from selected countries demonstrate the role of pharmacists in contributing to increased access to self-intervention for SRH.

### Data collection methodology

A comprehensive review of the literature was taken using multiple electronic databases: PubMed, Medline and grey literature. Data were also collected from the online websites of the public health ministries.

## The Egyptian experience

In Egypt, the women’s health programme established by the Ministry of Health (MOH) conducts family planning awareness campaigns during which SRH services are offered to young and adolescent girls as well as older women. These services include antenatal care, safe delivery, breastfeeding, immunization and premarital examination (a health assessment offered to soon-to-be married couples that includes tests for genetic, infectious and transmissible diseases and HIV testing). HIV/AIDS is still rare in Egypt; the prevalence of AIDS in Egypt is less than 0.1%. However, cases could be unreported or not investigated. This very low percentage might be due to Egypt’s conservative culture and the population’s lack of knowledge about HIV/AIDS [13]. In matters related to maternal health, the percentage of medically assisted births was 92% in 2014, and 90% of mothers received antenatal care from a trained provider [14].

The Egyptian health care system consists of two sectors: public and private. In general, the public health care system maintains low standards due to a lack of funding and poor staffing levels. Although public health care is provided free of charge in Egypt, many Egyptians avoid using these services for various reasons including the long waiting times for treatments, limited medical capacity and financial burden. A substantial portion of the population cannot afford frequent visits to the physician and only visit these health facilities in extreme situations such as serious illnesses and diseases. On the contrary, pharmacists who offer their high-quality knowledge and expertise at any time and free of charge are considered a cornerstone of the health care system. These free-of-charge consultations have a very positive impact on individuals’ out-of-pocket expenditure on health services and allow consultations on self-care interventions to be available across the entire nation [15]. For instance, around 75 000 community pharmacies serve patients 24 hours a day, 7 days a week (24/7).

In Egypt, topics and problems related to SRH are considered sensitive subjects, and female patients usually feel more comfortable when communicating with a female pharmacist to obtain self-care SRH consultation [16]. In Egypt, as in many EMR countries, medical products such as menstrual hygiene products, pregnancy tests and condoms can easily be obtained over the counter. Oral contraceptive pills, emergency contraceptive pills and intrauterine devices (IUDs) are also available over the counter [17, 18]. The easy access to these different choices of SRH medicines and devices helps individuals, especially youth, in their sexual health decisions.

During the height of the COVID-19 pandemic, Egyptian pharmacists supported patients in self-care interventions for both acute and chronic diseases. Pharmacists also supported patients by delivering counselling on the proper use of medications and devices used in sexual health. In some rural areas, pharmacists were vital for isolated patients, especially in the follow-up of cases [19]. Additionally, all private medical clinics were closed during that time, and patients were not able to find any health care professionals available to consult except the pharmacists [20]. Thus, many pharmacists offered free consultations via social media platforms such as Facebook, Twitter and WhatsApp. These free consultations answered patients’ varied questions about medicine and self-care interventions for SRH, among other health topics. Notifications and reminders were sent via SMS and email to patients to improve medication adherence, promote a healthy lifestyle and clarify to concerned patients that COVID-19 is not an STD. Egyptian pharmacists offered these types of services as a response to social distancing and quarantine measures. In fact, these services were not offered by pharmacists before the pandemic. The role of the Egyptian pharmacists during the pandemic is very important and was highlighted during the quarantine of the public, government and other health care providers [21]. These new methods of service delivery support health care services such as general self-care interventions, while providing a particular focus on SRH. Moreover, these new types of service delivery have established new channels of communication and trust between the pharmacists and the patients. The pharmacists are available 24/7 and give the patients a sense of security and relief knowing that their community pharmacist is accessible all the time.

Egyptian pharmacists are facing many challenges to providing their free services 24/7 since remuneration for counselling is not available in Egypt. This situation leads to disappointed pharmacists and forces them to limit these services when lack time exists. Moreover, physicians and pharmacists act as adversaries rather than members of the same team. This environment of competition within the medical community makes interprofessional collaboration difficult to consistently achieve [22].

## The Jordanian experience

Maternal and child health services have steadily improved in Jordan over the past few decades, while fertility has steadily declined, from 5.6 children in 1990 to 2.7 children in 2017–2018. However, the influx of Syrian refugees (with an average fertility rate of 4.7 children) into Jordan has placed a significant strain on the country’s health system, health providers and facilities, most notably on SRH services [23]. In fact, the unmet need for family planning is a major barrier to the development and empowerment of vulnerable segments of the local population, particularly citizens below the poverty line and Syrian refugees [24].

An ongoing project, the harmonized Reproductive Health Registry (hRHR), conducted by the Global Health Development/Eastern Mediterranean Public Health Network (GHD/EMPHNET) in collaboration with the MOH, aims to establish an hRHR in the Mafraq Governorate (the second largest governorate, which hosts the largest ratio of Syrian refugees in the Hashemite Kingdom of Jordan) in order to improve maternal and child health. The establishment of the hRHR will result in improved quality and timeliness of reproductive health data, better integration and interoperability among different health facilities, a strengthened referral system, increased capacity for decision-makers and providers to use health data, and cost plans to scale up the hRHR effort to other parts of the country [25].

In November 2019, health information was analysed and revealed the following [26]:

1. The current SRH information continues to be inadequate.
2. Women benefitting from SRH services still see the current SRH information system in the country as “insufficient and inaccurate”: Significant SRH services are not reported in either paper or electronic-based format.
3. There is a lack of reliable data, which limits the ability of health providers, planners and community leaders to make informed decisions.

In Jordan, the MOH works in coordination with the Royal Medical Services, university hospitals, private hospitals and a number of nongovernmental organizations (NGOs), including the United Nations Relief and Works Agency for Palestine Refugees in the Near East (UNRWA) and the Jordan Association for Family Planning and Protection (JAFPP), private physicians and pharmacies, to provide family planning methods [27].

During the COVID-19 pandemic, the Jordan Pharmacists Association (JPA) was the first health care provider body to recognize the important role of pharmacists in supporting the health care system during the pandemic and to take action on their behalf as follows:

- Pharmacies were exempted from the complete lockdown decision imposed by the government’s defense act, and accordingly community pharmacists were provided with special permits to resume the opening of their pharmacies [28].
- JPA pledged to disseminate drugs and medical devices, including home delivery, provided that it was done under the pharmacist’s direct supervision (despite this service normally being prohibited by drug and pharmacy law). After coordination with the Crisis and Management Health Team, JPA managed to obtain a letter of approval from the Jordan Food and Drug Administration (JFDA) [29]. This practice was stopped after relaxing of the country’s total lockdown and the gradual return to normal life [30].
- New delivery guidelines were put in place and circulated to all concerned community pharmacists to ensure proper dispensing by the pharmacist and to avoid cross-contamination [30].
- In collaboration with the digital economy and entrepreneurship ministry, JPA released a “Hello Pharmacist” telephone service for the public enquiring about the nearest available open pharmacy [30].
- The supply chain of medicines and medical supplies was maintained.
- JPA circulated messages through different social media platforms and interviews to instill public confidence regarding the availability of medicines supply.

The pandemic gave rise to several challenges in Jordan. The impact of the lockdown measures and the suspension of economic activities led to partial or entire income loss, medical supply (personal protective equipment [PPE]) shortages, and struggles to meet basic needs and accessible health care. However, private–public cooperation and openness transformed several challenges into opportunities. The pharmaceutical industry managed to produce safe-quality PPE to meet the country’s needs as well as for export to other countries. JPA has also successfully employed technology to maintain medical supply and health care services to the people in need [31].

## The Lebanese experience

As in many EMR countries, Lebanon remains conservative in many dimensions, especially with topics related to sex and sexuality. Attitudes regarding these topics are controlled by social, religious, gender and educational norms; they have yet to develop towards more equality and inclusivity. Statistics show that the maternal mortality ratio is 15 per 100 000 live births, with 0% of maternal deaths being attributed to HIV. The percentage of demand for family planning satisfied with a modern method of contraception is currently at 52.8%, with 78.8% of Lebanese having last used a condom during sexual intercourse. In fact, Lebanon has the lowest fertility rate among Arab countries, with a current total fertility rate (TFR) of 1.9. Data are not available for the percentage of patients with syphilis or for pregnant women who know their HIV status [32]. Assi et al. concluded that with the rising prevalence of HIV and sexually transmitted infections (STIs) locally in Lebanon and in the EMR as a whole, there is an urgent need for the promotion of sexual health education and services [33]. Other studies have reported that Lebanese youth are exposed to unsafe sexual practices, leading to unplanned pregnancy, STIs and abortion [34]. Lack of information and education on HIV/AIDS and STDs, especially for young adults, as well as a lack of confidentiality, privacy and proper counselling, has pushed the Ministry of Public Health (MOPH) to affirm a serious commitment to improving SRH in Lebanon by establishing many policies and programmes, including supporting HIV-affected individuals and family planning programmes. In April 2012, Lebanon initiated youth-friendly services including SRH services at a limited number of delivery service points [35]. An interactive website on SRH for young people has also been developed. Another programme, the revised version of the service delivery guidelines for reproductive health, was launched in 2015 [35]. This programme was aimed at improving the knowledge, skills and confidence of health workers to ensure that the reproductive health needs of women, men and youth are met in times of stability and crisis. Reproductive health medications and supplies are provided through primary health care centres (PHCs). Equipment that enables early detection of reproductive system diseases (such as breast and cervical cancer) is also provided. Reproductive health information is also delivered to beneficiaries [36]. Additionally, since 1989, the MOPH has funded a national AIDS programme. This programme aims to fight HIV/AIDS, and includes all kinds of activities, from prevention and treatment to care and support, with a special focus on the prevention of mother-to-child transmission and on the most at-risk population. Today, more than 110 voluntary counselling and testing (VCT) centres are located throughout the country, and services are being delivered by trained health care workers and nurses [37]. Stakeholders of the national AIDS programme comprise many public and private organizations, including nonprofit organizations, universities and sexual health centres.

Despite the importance of community pharmacies as one of the easiest health care points where individuals – notably those who are most vulnerable, such as youth and women – can be sensitized to SRH and have access to educational and prevention services, pharmacists are still excluded from all SRH national programmes. Pharmacists do not benefit from the training or the support delivered to other primary health care workers. MOPH campaigns are not run in the community, and pharmacists to this day and are yet to be acknowledged as important key players in SRH interventions, notably in empowering individuals for self-care interventions.

Nevertheless, many studies conducted in Lebanon showed the pharmacists’ fundamental and effective role in SRH. Pharmacies are the single most important source of contraceptives [38], followed by dispensaries, nurses and midwives [39]. In the Jurjus study, 88.4% of army soldiers indicated a pharmacy as the source of condoms [40]. Tannouri revealed an ample need for information related to SRH, especially among the youth category, showing a greater need for knowledge related to STDs and safe sexual practices [41, 42]. In the Jurjus study, when army soldiers were faced with an infection, 39.6% had chosen to take no action. Only 41.4% had consulted a health professional and 5.4% a pharmacist [40]. Easy pharmacy access makes SRH commodities available for these individuals who are unwilling or unable to receive these services from another health care centre or provider.

Pharmacists with solid scientific knowledge and ability in terms of patient counselling serve as reliable health care providers offering a private and comfortable discussion with patients about their SRH needs and inquiries [43]. They work to support and empower patients with regard to different self-care interventions for SRH, whether for fertility management, contraception or proper use of diagnostic products for STDs. For instance, female patients are able to ask and learn how to properly use ovulation predictors at home at the precise time of their fertile window to better plan intercourse and increase chances of conception. On the other hand, patients seeking contraception will be able to choose between different contraceptive methods, select the optimal method for their specific circumstances, and learn how to properly use this contraceptive method (especially oral contraceptives). The patient will be able to handle side effects, missed doses, and the proper use of at-home emergency contraception. Individuals willing to know their HIV status can procure an HIV self-test kit in their community pharmacy, and with the support of their pharmacist will be able to correctly do the test at home and learn how to interpret the results.

During the COVID-19 pandemic, pharmacists were exempted from nationwide house confinement. Thus, pharmacies were open all the time; patients have continued to seek their help and services with no interruption [44]. Community pharmacists offered individual initiatives such as free teleconsultation through different platforms, including WhatsApp messages or phone calls. The movement from face-to-face counselling to virtual counselling was very helpful for the continuity of care and support, as patients were still able to be proactive about their SRH choices. This has been reflected in their quality of life during pandemic times.

The challenges facing pharmacies have been numerous, primarily as a result of Lebanese laws and regulations forbidding the home delivery of medications as well as pharmacist vaccination delivery, even during the time of crisis. Other obstacles could be the lack of cooperation between the community pharmacists and other primary health care providers, especially family physicians and general practitioners.

Finally, the lack of physicians and government support, as well as their lack of recognition of the crucial and fundamental role of community pharmacists, could influence the time and efforts dedicated by the Lebanese pharmacists to educate and train patients on self-care interventions, notably for SRH.

## The Somali experience

SRH indicators in Somalia are among the worst in the world [45], with very limited availability of specific data. With the exception of HIV-related cases, SRH services have become less stigmatic and more readily available and affordable. Self-care interventions for SRH can be accessed through several health care centres, including hospitals and community pharmacies. Community pharmacy personnel have been reported as the primary health care destination for more than two decades in the country. Some of the public and private hospitals have provided similar services in the past decade. People do not voluntarily seek HIV diagnosis and treatment because of HIV stigma, discrimination and the resultant fear of being identified as HIV-positive. HIV stigma and social rejection limit the disclosure of one’s HIV status to families and communities; this situation is worse in vulnerable internally displaced and rural areas of the country [46]. Many campaigns have been conducted in both the public and private health sectors. For instance, the “Bridging the Gap” campaign was initiated in observance of World AIDS Day, targeting what is considered to be the most vulnerable group in Somalia, the youth. Approximately 200 university students received free HIV screening at Banadir Hospital through this campaign [47]. For the last 8 years, humanitarian efforts have been directed towards MOH centres where protection and other public health services are provided to vulnerable women and HIV-positive individuals. An example of these services is the provision of self-care items such as soap and menstrual hygiene/sanitary pads to women and girls [48]. Additionally, as part of their curriculum, universities are conducting multiple public health campaigns, especially on topics considered as taboo such as SRH. Furthermore, a number of the largest hospitals are offering programmes for women from different economic and educational backgrounds – programmes promoting free SRH testing such as HIV tests. The Somali Pharmacists Association (SPA) has developed a system of registering pharmacists with active international pharmacy licenses. More than 90 registered pharmacists are working across the country in the public and private sectors, and are more involved in delivering and administering sexual and reproductive medicines and devices compared to health care centres. For instance, pharmacists deliver different contraceptive methods including oral contraceptive pills, IUDs or condoms directly to the patients. They also offer vaginal cleansers and topical anti-infective treatments.

During the COVID-19 pandemic, Somali pharmacists offered counselling on self-care for SRH services and activities via social media platforms as well as by calling patients directly on the phone. According to authors’ findings, services included providing customized treatments for COVID-19 patients, especially women and children. Pharmacists were ready to respond to any inquiries from the community and provide pharmaceutical consultations accordingly. One of the most important areas in which pharmacists were engaged was self-care interventions for minor ailments, infection control and eradication, and STI testing and treatments.

During COVID-19, one of the challenges faced in Somalia has been the absence of education among community pharmacists regarding the pandemic, and more importantly, the supportive treatment measures. The failure to identify supportive care medicines alleviating COVID-19 symptoms was the main issue with pharmacy personnel despite the drugs being available in their pharmacies. The physicians and most of the pharmacists were not updated regarding treatment options, especially the appropriate and rational antibiotic therapy in the case of superinfection. Lack of guidelines and policies for the supportive treatments of COVID-19 pushed many patients to self-medicate. This resulted in drug–drug interactions and reduced effectiveness. Despite coping with the COVID-19 crisis, community pharmacists did not prioritize patient education, training and counselling on SRH self-care interventions. This has compromised the belief of the community regarding the relation between SRH diseases and COVID-19.

## The real impact of pharmacists in a crisis: the COVID-19 pandemic case

The COVID-19 pandemic has had a profound effect and led to severe disruption for the global health and development community in all aspects. More than half (53%) of countries have experienced partially or completely disrupted services for hypertension treatment, 49% for treatment for diabetes and diabetes-related complications, 42% for cancer treatment and 31% for cardiovascular emergencies. Rehabilitation services have been disrupted in almost two thirds (63%) of countries [49].

Self-care interventions are important during a pandemic such as COVID-19. Major disruptions to the normal functioning of national health systems have created the need to respond to people who have been or are affected by the virus, with evidence-based, high-quality self-care interventions. These interventions can provide an important alternative to the usual health facility or health worker-based services. When provided the opportunity to offer their clinical expertise, pharmacists have a major role in empowering and supporting patients through education and dissemination of health information by all means at their disposal. The outcomes of their utilization are positive economic figures, fewer medical complications, and improved therapeutic results. Their importance is much more recognized in areas where there is a severe shortage of other health care professionals, pharmacists and pharmaceutical personnel than in areas where there is greater availability to provide care.

Digital technologies and platforms such as help lines through the phone, websites or social media that have recognized expertise may be available for consultation or follow-up. Other telehealth services outside the traditional pharmacy visit could include patient consultations using video conferencing, teleconferencing and/or chat rooms, as well as remote patient monitoring of vital signs, point-of-care testing and other results [50]. When utilizing digital health tools, pharmacists can interpret patients’ vital signs, continue patient care at home, perform medication reconciliation and inspire patients to be more productive and consistent with their health care. Under constrained financial and economic conditions, along with a lack of health care staff, digital and mobile health can help reach people in rural areas.

### Considerations for a post-COVID-19 scenario

Pharmacists have shown and are still showing their preparedness during public health dangers and crises. One of their advantages is their accessibility, which is rare among health care professionals during these times. Their role has been highlighted during the COVID-19 pandemic, especially when all other health care providers were shut down and hospital admissions were prioritizing pandemic patients. During shortages of health care providers, pharmacists can offer alternatives to support patients and find creative solutions to empower them.

The COVID-19 pandemic emphasizes the importance of pharmacists’ expertise in training and educating individuals on self-care interventions in general and on SRH in particular. Pharmacists are challenged to provide their education and counselling while effectively protecting themselves. For all these reasons, pharmacists must adapt quickly, train themselves and interact with patients using innovative new technologies. Empowering pharmacists to become leaders of digital health solutions within and beyond their practice settings is becoming a necessity during these pandemic times [50].

The COVID-19 pandemic has highlighted the value of having well-trained modern pharmacists able to adapt to any new modalities in patient education and services. For this purpose, it is essential to incorporate self-care interventions in general and SRH in particular into the curriculum of schools of pharmacy. The curriculum should include a module on SRH with specific learning objectives to train each pharmacy student to be fully equipped with the information needed on SRH to practice in the community. Modules can also include live training in the community.

“Students Today, Pharmacists Tomorrow” is the slogan of the International Pharmaceutical Students’ Federation (IPSF), where students today will learn how to educate patients on SRH topics such as safe sex, methods of contraception, pregnancy and management of STIs. In return, future pharmacists will carry the message learned and serve as a catalyst in the community to spread awareness and empower patient self-care.

A change or strengthening of policies at the national level would allow for the effective use of the defined skills and competencies of the pharmacy profession in disseminating, introducing and scaling up access to self-care interventions for SRH. Pharmacists should be included as essential role players in the process of developing policies promoting and protecting health and well-being.

There is a need for a policy statement between FIP and WHO on improving self-care service delivery mechanisms.

## Conclusion

Pharmacists play a vital role in promoting self-care interventions for SRH in the EMR. Inequalities in access to SRH information and services continue to be one of the greatest challenges in these countries, and especially in LMICs. EMR pharmacists offer their high-quality knowledge and expertise at any time, free of charge and in complete privacy. They should be acknowledged as a cornerstone of the health care system. The increase in self-care interventions during pandemics highlights the importance of empowering individuals when access to health care systems and professionals is difficult.

During the COVID-19 pandemic, EMR pharmacists supported patients in self-care interventions. Many offered free consultation via social media platforms such as Facebook. Others sent notifications and reminders to improve patients’ medication adherence, as well as healthy living advice by SMS and email. WHO has an important role in establishing the standardization and the guidelines for self-care interventions for SRH. This was stated in the *WHO consolidated guideline on self-care interventions: sexual and reproductive health and rights* issued in 2019. FIP has an essential role in leading pharmacists in the implementation of these guidelines: this evidence-based normative guidance will result in the successful implementation in the national context and incorporation within national policies. The governments in Egypt, Jordan, Lebanon and Somalia have created and implemented different programmes to promote and encourage self-interventions for SRH. However, the pharmacists in these countries are still not involved in these programmes, and more governmental support and acknowledgment of the pharmacist’s role is vital. Nevertheless, pharmacists in the Middle East are considered trusted health care professionals from various points of view, based on their education, credibility and expertise in providing necessary health care services to the public in areas where conventional providers are lacking, particularly in SRH care.

## Data Availability

Data sharing is not applicable to this article as no data sets were generated or analysed during the current study.

## Abbreviations

EMR: Eastern Mediterranean Region
SRH: Sexual and reproductive health
COVID-19: Coronavirus disease 2019
WHO: World Health Organization
LMICs: Low- and middle-income countries
STDs: Sexually transmitted diseases
FIP: International Pharmaceutical Federation
UNFPA: United Nations Population Fund
YPS: Youth peace and security
HIV: Human immunodeficiency virus
AIDS: Acquired immunodeficiency syndrome
IUDs: Intrauterine devices
24/7: 24 hours a day, 7 days a week
GSCF: Global Self-Care Federation
HPV: Human papillomavirus vaccine
GHD/EMPHNET: Global Health Development/Eastern Mediterranean Public Health Network
hRHR: Harmonized Reproductive Health Registry
MOH: Ministry of Health
MNCH: Maternal, neonatal and child health
NGOs: Nongovernmental organizations
UNRWA: United Nations Relief and Works Agency for Palestine Refugees in the Near East
JAFPP: Jordan Association for Family Planning and Protection
JPA: Jordan Pharmacists Association
JFDA: Jordan Food and Drug Administration
PPE: Personal protective equipment
TFR: Total fertility rate
STIs: Sexually transmitted infections
PHC: Primary health care
MOPH: Ministry of Public Health
VCT: Voluntary counselling and testing
OTC: Over the counter
